# Elemene Injection Induced Autophagy Protects Human Hepatoma Cancer Cells from Starvation and Undergoing Apoptosis

**DOI:** 10.1155/2014/637528

**Published:** 2014-07-24

**Authors:** Yan Lin, Keming Wang, Chunping Hu, Lin Lin, Shukui Qin, Xueting Cai

**Affiliations:** ^1^Institute of First Clinical Medicine, Nanjing University of Chinese Medicine, Nanjing, Jiangsu 210046, China; ^2^Department of Oncology, The Second Affiliated Hospital of Nanjing Medical University, Nanjing, Jiangsu 210021, China; ^3^Laboratory of Cellular and Molecular Biology, Jiangsu Province Institute of Traditional Chinese Medicine, 100 Shizi Street, Hongshan Road, Nanjing, Jiangsu 210028, China; ^4^Oncology Center of Chinese, 81 Hospital of PLA, Nanjing, Jiangsu 210002, China

## Abstract

Elemene, a compound found in an herb used in traditional Chinese medicine, has shown promising anticancer effects against a broad spectrum of tumors. In an *in vivo* experiment, we found that apatinib, a tyrosine kinase inhibitor that selectively inhibits VEGFR2, combined with elemene injection (Ele) for the treatment of H22 solid tumor in mice resulted in worse effectiveness than apatinib alone. Moreover, Ele could protect HepG2 cells from death induced by serum-free starvation. Further data on the mechanism study revealed that Ele induced protective autophagy and prevented human hepatoma cancer cells from undergoing apoptosis. Proapoptosis effect of Ele was enhanced when proautophagy effect was inhibited by hydroxychloroquine. Above all, Ele has the effect of protecting cancer cells from death either in apatinib induced nutrient deficient environment or in serum-free induced starvation. A combination of elemene injection with autophagy inhibitor might thus be a useful therapeutic option for hepatocellular carcinoma.

## 1. Introduction

Primary liver cancer is the most common malignant tumor, which accounts for 45% of the global incidence of the morbidity and mortality rates are in the second tumor spectrum. Hepatocellular carcinoma (HCC), a primary liver cancer, is the most common type accounting for 90% of primary liver cancer. In our country the survival time of patients with advanced HCC is typically only 3–6 months. Elemene is extracted from traditional Chinese medicine* Curcuma zedoaria (Christm.) Rosc*.* In vivo* and* in vitro* experiments confirmed elemene injection (Ele) has good induction of apoptosis and antiangiogenic effects on a variety of tumor cells [1, 2]. In this study, we found elemene injection had both proapoptotic and proautophagic effect on human hepatoma cell line HepG2. Since autophagy can result in both survival and cell death, we then asked whether Ele-induced autophagy is protective or proapoptotic. The results revealed that Ele elemene injection induced autophagy protects human hepatoma cancer cells from starvation and undergoing apoptosis.

## 2. Materials and Methods

### 2.1. Materials

Elemene injection was obtained from Dalian Holley Kingkong Pharmaceutical (Dalian, China). Apatinib was provided by Jiangsu Hengrui Medicine Co., Ltd. Hydroxychloroquine (HCQ) was purchased from Jiangsu Province Hospital on Integration of Chinese and Western Medicine, China. MTT (3-(4,5-dimethylthiazol-2-yl)-2,5-diphenyltetrazolium bromide) was obtained from Sigma, USA. Lysis buffer was purchased from Beyotime, China. Antibodies (PARP, cleaved-caspase 3, cleaved-caspase 9, Bcl-2, and Bax) were obtained from Cell Signaling Technology, USA. Anti-LC3 antibody was purchased from Abcam plc, USA. GAPDH antibody was purchased from Santa Cruz, USA. IRDye 800CW goat anti-rabbit IgG (H + L) and IRDye 680RD goat anti-mouse IgG (H + L) were obtained from LI-COR Biosciences, USA.

### 2.2. Cell Lines and Animals

Human hepatoma cell line HepG2 was purchased from the Cell Bank of Shanghai Institute of Biochemistry and Cell Biology, Shanghai Institutes for Biological Sciences, Chinese Academy of Sciences. Cells were cultured in DMEM medium supplemented with 10% fetal bovine serum (FBS), 100 U/mL penicillin, and 100 *μ*g/mL streptomycin (all available from Invitrogen, Grand Island, NY, USA). All cultures were maintained in a humidified environment with 5% CO_2_ at 37°C. Mouse hepatoma H22 cells were maintained in the peritoneal cavities of ICR mice provided by KeyGEN Biotech (Nanjing, China). ICR mice were purchased from Shanghai SLRC Laboratory Animal Co., Ltd. (Shanghai, China). Mice were housed under specific pathogen-free conditions and provided with a standard rodent laboratory diet from Shanghai SLRC Laboratory Animal Co., Ltd. All experimental protocols were approved by the Animal Care and Use Committee of Jiangsu Branch of China Academy of Chinese Medical Sciences and were in accordance with the Declaration of the National Institutes of Health Guide for Care and Use of Laboratory Animals (Publication no. 80-23, revised 1996).

### 2.3. Cytotoxicity Assay

The cytotoxicity of Ele on HepG2 cells was analyzed by MTT assay. HepG2 cells at mid-log phase were seeded in 96-well plate at a density of 1 × 10^4^ cells per well in 100 *μ*L medium. After 24 h incubation, cells were exposed to DMEM (used as control in all experiments), 0.05, 0.1, 0.15, or 0.2 mg/mL Ele for 24 and 48 h. After treatment, 10 *μ*L of 5 mg/mL MTT was added and the cells were incubated for 4 h at 37°C. The supernatant was discarded and 100 *μ*L of DMSO was added to each well. The mixture was shaken on a minishaker at room temperature for 10 min and the spectrophotometric absorbance was measured by Multiskan Spectrum Microplate Reader (Thermo, USA) at 570 nm and 630 nm (absorbance 570 nm, reference 630 nm). Triplicate experiments were performed in a parallel manner for each concentration point and the results were presented as mean ± SD. The net OD_570 nm_ − OD_630 nm_ was taken as the index of cell viability. The net absorbance from the wells of cells cultured with DMEM was taken as the 0% inhibitory rate. The percent inhibitory rate (IR%) of the treated cells was calculated by the following formula:

(1) $$\begin{array}{c} \text{IR}\%=1-\frac{{\left({\text{OD}}_{570\;\text{nm}}-{\text{OD}}_{630\;\text{nm}}\right)}_{\text{treated}}}{{\left({\text{OD}}_{570\;\text{nm}}-{\text{OD}}_{630\;\text{nm}}\right)}_{\text{control}}}\times 100\%. \end{array}$$

### 2.4. Apoptosis Assay

HepG2 cells were cultured, including positive and negative controls, for appropriate time to induce apoptosis. Apoptotic cells were determined with Guava Nexin Reagent (Millipore, USA) according to the manufacturer's protocol. Briefly, the cells were harvested and resuspended with 100 *μ*L DMEM medium containing 1% FBS. The cells were then incubated for 20 min at room temperature in the dark with 100 *μ*L of Guava Nexin Reagent containing Annexin V-PE and 7-AAD. Afterward, apoptosis was analyzed by flow cytometer (Guava 6HT, Merck-Millipore, USA). The data were analyzed using the software Guava 2.5.

### 2.5. Western Blotting Analysis

For preparation of cell extracts, cells were treated as described in the figure legends and lysed with lysis buffer (Beyotime, China) on ice. Lysate was centrifuged at 13,000 g for 5 min at 4°C. The concentration of protein in the supernatants was detected by Nanodrop 1000 Spectrophotometer (Thermo, NH, USA). Equal amount of protein was separated on 13% SDS-polyacrylamide gels (SDS-PAGE) and transferred onto the PVDF membranes (Millipore, MA, USA). After being blocked with 1% BSA in TBST (Tris Buffered Saline with Tween-20) for 2 h, membranes were incubated with primary antibodies overnight at 4°C. Blots were washed and incubated with secondary antibodies for 2 h at room temperature. Membranes were again washed three times with TBST. Detection was performed by LI-COR Odyssey Scanner (LI-COR Biosciences, USA).

### 2.6. Evaluation of Antitumor Activity of Ele Combined with Apatinib

H22 hepatoma cells were maintained in the peritoneal cavities of ICR mice. 20 ± 2 g ICR mice, half male and half female, were inoculated subcutaneously (s.c.) 0.2 mL H22 ascites tumor cell suspension (about 5 × 10^6^/mL). Treatment was started the day after. The inoculated mice were randomized into six groups (*n* = 9): 200 mg/kg apatinib group, 75 mg/kg Ele group, 50 mg/kg Ele + 200 mg/kg apatinib group, 75 mg/kg Ele + 200 mg/kg apatinib group, 100 mg/kg Ele + 200 mg/kg apatinib group, 75 mg/kg Ele + 100 mg/kg apatinib group, 75 mg/kg Ele + 300 mg/kg apatinib group, and H22-bearing mice as negative control group (NS group). Ele was administered i.p. once daily while apatinib was used by intragastric injection administration once daily. Tumor size was measured using a caliper across its longest diameter (a) and the second longest diameter (b), and its volume was calculated using TV = 0.5 ab^2^. Inhibition ratio (IR) was calculated using IR(%) = {(TV_control_ − TV_treat_)/TV_control_} × 100. All the mice were sacrificed after 8 days of treatment.

### 2.7. Statistical Analysis

All the data were expressed as mean ± standard deviation (SD). Statistical analysis was performed using the Student's *t*-test and *P* < 0.05 was indicated to be statistical significance.

## 3. Results

### 3.1. Ele Suppressed the Proliferation of Hepatoma Cancer Cells HepG2

**Figure 1:**
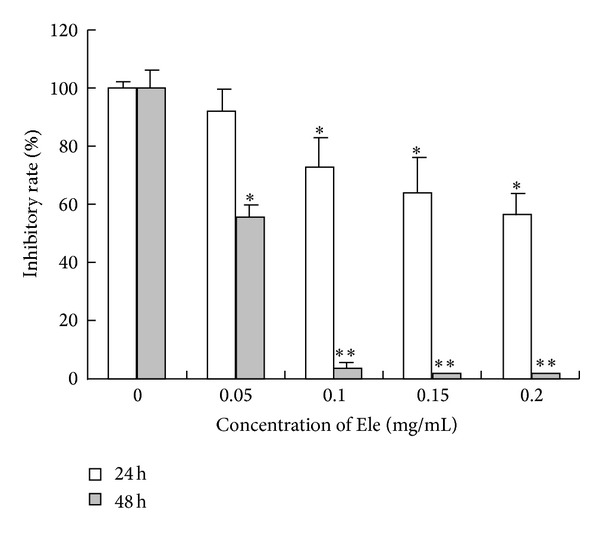
Cytotoxicity effect of Ele on HepG2 cells. The results shown were the mean of three parallel experiments (triplicate wells) for each concentration point (0.05, 0.1, 0.15, and 0.2 mg/mL) at 24 and 48 h. *P < 0.05, **P < 0.01 versus control.

**Figure 2:**
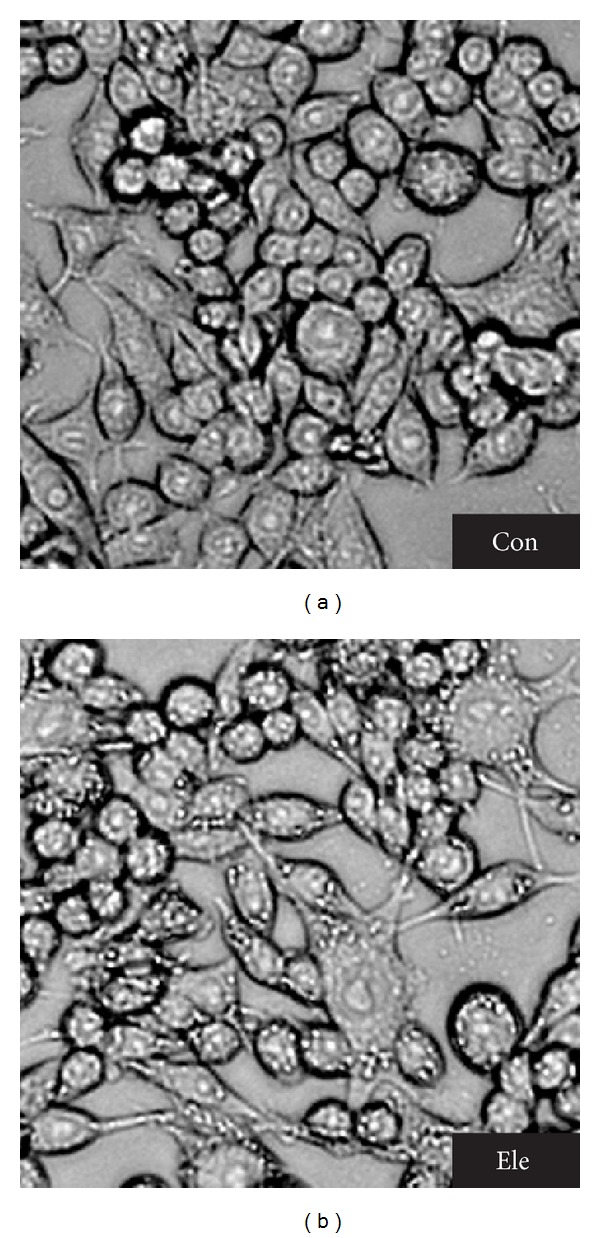
Cell morphological assessment. Light micrograph of HepG2 cells after overnight incubation with Ele. Compared to the control cells, cells exposed to Ele presented typical autophagy morphology with marketable vacuoles (×200).

MTT assay was applied to analyze the inhibition effect of Ele on cell growth.  Figure 1 showed that Ele inhibited proliferation of HepG2 cells in a dose-dependent, time-independent manner. Interestingly, marketable vacuoles in the cytoplasm of Ele-treated cells were observed under an inverted light microscope (Figure 2). However, this phenomenon was not observed in negative control.

### 3.2. Ele Induced Both Cell Apoptosis and Autophagy on HepG2 Cells

**Figure 3:**
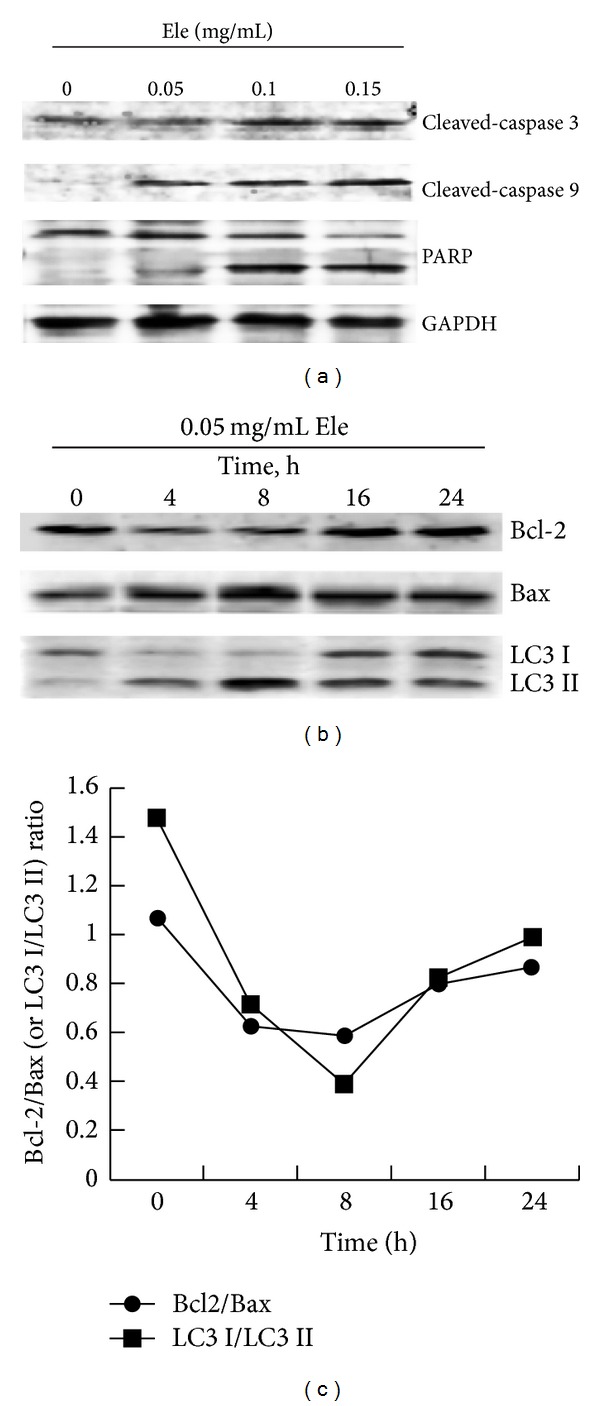
Ele-induced apoptosis and autophagy of HepG2 cells. HepG2 cells were untreated or treated with different concentration of Ele for 24 h or with 0.05 mg/mL Ele for different times. Apoptosis-related proteins (a) and autophagy-related proteins (b) were detected by Western blotting. The Bcl-2/Bax protein ratio and LC3 I/LC3 II protein ratio were shown in (c).

Apoptosis was controlled by regulators, which either have an inhibitory effect on programmed cell death (antiapoptotic) or block the protective effect of inhibitors (proapoptotic) [3, 4]. After treatment with different concentrations of Ele for 24 h, a concomitant increase in the levels of cleaved-caspase 3, cleaved-caspase 9, and cleaved poly-ADP-ribose polymerase (PARP) was observed (Figure 3(a)), which indicted that Ele-induced cell apoptosis was a mitochondrial (intrinsic) pathway. HepG2 cells, which were exposed to Ele for different times, were lysed and prepared to detect the antiapoptotic proteins (Bcl-2) and proapoptotic protein (Bax). As shown in Figure 3(b), the expression of Bax was increased and the expression of Bcl-2 was decreased after 4 h and 8 h of treatment with 0.05 mg/mL Ele.

LC3 protein is a biochemical marker for autophagic cells [5]. To confirm that Ele also can induce autophagy on HepG2 cells, Western blotting was used to detect the impact of Ele on the cleavage of protein LC3. LC3 has two forms, LC3-I and LC3-II. The latter is located on the autophagosomes and always remains on the membrane. LC3-II is considered as the sign molecule of autophagy. As shown in Figure 3(b), exposure to Ele for different times, expression of LC3 I protein reduced while that of LC3 II increased at 4 h and 8 h time points, indicating that Ele could induce autophagy on HepG2 cells.

The Bcl-2/Bax protein ratio and LC3 I/LC3 II protein ratio were shown in Figure 3(c), during 0–8 hours, Bcl-2/Bax protein ratio gradually decreased with time course, indicating that the degree of cell apoptosis increased, and LC3 I/LC3 II protein ratio gradually decreased with time course, indicating that the degree of autophagy increased. But during the 16–24 hours, Bcl-2/Bax protein ratio and LC3 I/LC3 II protein ratio began to increase, indicating that cell apoptosis and autophagy began to reach a balance. Did autophagy protect the cell from dying via inhibiting cell apoptosis?

### 3.3. Ele Protected HepG2 Cells during Starvation

**Figure 4:**
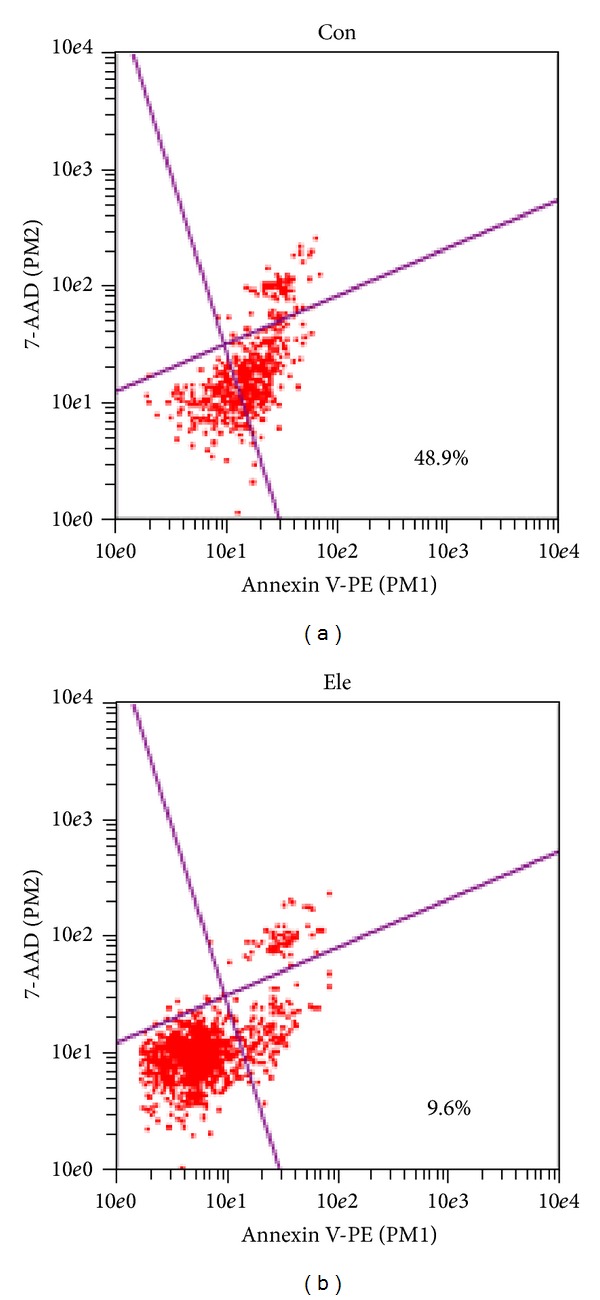
Ele protected HepG2 cells during starvation. HepG2 cells, starved for 12 h, were untreated or treated with 0.05 mg/mL Ele for 24 h. Flowmetry was used to detect the apoptosis cells.

HepG2 cells were starved with serum-free medium for 12 h than treated with either control (DMEM) or 0.05 mg/mL Ele. Flowmetry assays showed that treatment with Ele significantly decreased cell apoptosis rate, compared with control group (9.6% versus 48.9%, *P* < 0.05) (Figure 4).

### 3.4. Ele Reduced the Effectiveness of Apatinib on H22 Solid Tumor in Mice

**Figure 5:**
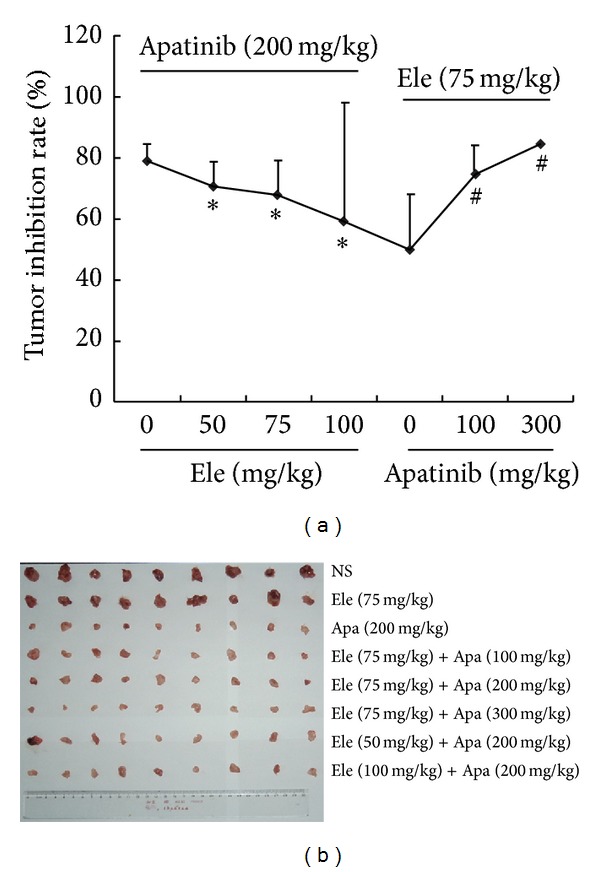
Ele reduced the effectiveness of apatinib on H22 solid tumor in mice. The H22 bearing mice were randomized into six groups (*n* = 9): 200 mg/kg apatinib group, 75 mg/kg Ele group, 50 mg/kg Ele + 200 mg/kg apatinib group, 75 mg/kg Ele + 200 mg/kg apatinib group, 100 mg/kg Ele + 200 mg/kg apatinib group, 75 mg/kg Ele + 100 mg/kg apatinib group, 75 mg/kg Ele + 300 mg/kg apatinib group, and H22-bearing mice as negative control group (NS group). All the mice were sacrificed after 8 days of treatment. Inhibition ratio (IR) was calculated using IR(%) = {(TV_control_ − TV_treat_)/TV_control_} × 100. *P < 0.05 versus apatinib, ^#^ *P* < 0.05 versus Ele.

Using H22 solid tumor model, the result showed that Ele combined with Apa reduced the effectiveness of Apa in a dose-dependent manner (Figure 5). Apatinib, also known as YN968D1, is a tyrosine kinase inhibitor that selectively inhibits the vascular endothelial growth factor receptor-2 (VEGFR2, also known as KDR). It is an orally bioavailable, small molecule agent which is thought to inhibit angiogenesis in cancer cells; specifically apatinib inhibits VEGF-mediated endothelial cell migration and proliferation, thus blocking new blood vessel formation in tumor tissue [6].

### 3.5. Ele-Induced Autophagy Protected HepG2 Cells from Undergoing Cell Apoptosis

**Figure 6:**
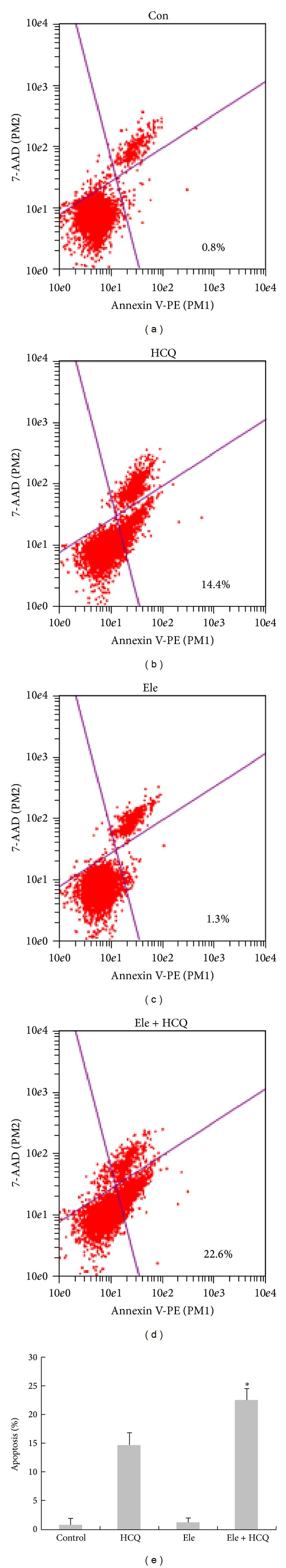
Ele-induced autophagy protected HepG2 cells from undergoing cell apoptosis. HepG2 cells, untreated or treated with either 0.05 mg/mL Ele or 50 *μ*M HCQ or cotreated with Ele and HCQ for 24 h, were harvested and detected by Flowmetry. *P < 0.05 versus HCQ group.

Since autophagy can result in both survival and cell death, we then asked whether Ele-induced autophagy is protective or proapoptotic. HepG2 cells were treated with either 0.05 mg/mL Ele or 50 *μ*M HCQ (a lysosomal alkalization agent could prevent autophagosome fusion with lysosomes, which is leading to increased accumulation of autophagosome, stopping autophagy at the late phase) or cotreated with Ele and HCQ for 24 h. Flowmetry assays showed that cotreatment with Ele and HCQ significantly decreased cell viability, compared with the cells treated with Ele alone (22.6 ± 1.9% versus 1.3 ± 0.7%, *P* < 0.05) (Figure 6).

## 4. Discussion

The treatment of advanced primary liver cancer is currently very poor due to the lack of effective treatment. An estimated 748,300 new liver cancer cases and 695,900 cancer deaths occurred worldwide in 2008, one of the first three cancer-related deaths [7]. Among primary liver cancers, hepatocellular carcinoma (HCC) represents the major histological subtype accounting for 70% to 85% of the total liver cancer burden worldwide [8]. At present, China still has the largest number of deaths and is the country of the highest prevalence, accounting for about 55% of the number of cancer cases worldwide and accounting for 45% of cancer deaths worldwide. Because liver cancer has insidious onset, rapid development, easy invasion, and easy transfer characteristics, patients have liver cancer which can be surgically removed is only 20%, while the majority of patients are found to have locally advanced or distant metastasis. HCC's prognosis is very poor; 5-year survival rate is less than 10% in Asian countries, 8% in Europe, and only about 5% in developing countries. Surgery and liver transplantation are currently the main treatment; 5-year recurrence rate after radical resection of hepatocellular carcinoma is still up to 60–70%; even in small HCC recurrence rate is also around 40–50% [9]. Due to limitations of liver transplantation in patients with hepatic sources, physical underlying diseases, and other factors, only a small number of people benefit. But even after a successful liver transplant there are some people who relapse and suffer from metastasis. Some patients will relapse even after a successful liver transplantation. Patients with middle and late stage primary liver cancer lost surgery and other local treatment opportunity, such as transcatheter arterial chemoembolization (TACE), microwave ablation (MWA), and percutaneous ethanol injection (PEI). Systemic chemotherapy for advanced hepatocellular carcinoma may have some effects and some patients' symptoms improved; however, many reported single-agent or combination chemotherapy effective rate is very low. Therefore, there is an urgent clinical need for an efficient and low toxicity treatment to improve survival in patients with advanced hepatocellular carcinoma. Elemene injection has better inhibitory role on tumor cells with low toxicity, mild side effects of liver, and kidney function, suitable for advanced hepatocellular carcinoma patients. In recent years, along with the fast development of traditional Chinese medicine, many modem Chinese herbal medicine were applied to clinical use. For example, ginsenosides Rg3 (Shenyi capsule) [10, 11], elemene injection [12–14], arsenic trioxide (As2O3) injection [15], Kanglaite injection, and Shenmai injection, which all have certain effect on inhibiting tumor cell proliferation and antitumor angiogenesis. Chinese materia medica preparation is characterized by certain antitumor effects; adverse reactions are mild, can enhance immunity, and improve the quality of life of patients, a wide range of applications, especially for advanced liver cancer which cannot be systemic chemotherapy or progression after chemotherapy, but the overall disadvantage of traditional Chinese medicines tumor is the fact that the cell killing effect is weak. Elemene injection is considered strongest Chinese medicine in killing tumor cells in the present study. Recent experimental studies showed that elemene injection has significantly cytotoxicity on a variety of tumor cells [12–14], antitumor metastasis, invasion, and inhibition of tumor angiogenesis effect. In this study, elemene monotherapy inhibited tumors proliferation in H22 hepatoma bearing mice, consistent with the above-mentioned literature.

Autophagy (or autophagocytosis) is the basic catabolic mechanism that involves cell degradation of unnecessary or dysfunctional cellular components through the actions of lysosomes [16]. Oftentimes, cancer occurs when several different pathways that regulate cell differentiation are disturbed. Autophagy plays an important role in cancer in both protecting against cancer and potentially contributing to the growth of cancer [17]. Autophagy may protect against cancer by isolating damaged organelles, allowing cell differentiation, increasing protein catabolism, and even promoting cell death of cancerous cells [18]. However, autophagy can also contribute to cancer by promoting survival of tumor cells that have been starved. Herman-Antosiewicz et al. [19] indicated that induction of autophagy represents a defense mechanism against sulforaphane-induced apoptosis in human prostate cancer cells. In addition, chemotherapy and radiotherapy increased autophagic activity, and autophagy cleared the ionizing radiation or cytotoxic induced damaged mitochondria, blocking the mitochondrial apoptotic signal transduction cascade so that some cancer became drug resistant [20–22]. Some autophagy inhibitors help improve the antitumor therapeutic effect. 3-Methyladenine (3-MA) and chloroquine (CQ) are two common autophagy inhibitors. 3-MA inhibits phosphoinositide 3-kinase (P13K) and can inhibit MAP1-LC3 I transfer to MAPl-LC3 and thus interfere with or block autophagy. HCQ can increase the pH value inside the lysosome and inhibit the fusion of autophagy and lysosome.

In the present study apatinib inhibited tumor blood vessels leading to nutritional deficiencies; in that state, elemene injection induced autophagy and degradation of proteins in tumor cells for nutritional supplements, thereby reducing the efficacy of apatinib. When combined with HCQ, an autophagy inhibitor, further enhances cell apoptosis in Ele-treated HepG2 cells. Does elemene still have existing proautophagy effect in other tumor cells? Does elemene still have existing antagonistic effect when combined with other drugs, such as cytotoxic drugs, due to induction of autophagy? All these issues need to be clarified by further research.
